# Marine microbial mock communities for validating rRNA gene amplicon sequencing

**DOI:** 10.1128/mra.00298-25

**Published:** 2025-05-30

**Authors:** Robert H. Lampe, Ariel J. Rabines, Bryce A. Ellman, Hong Zheng, Andrew E. Allen

**Affiliations:** 1 Integrative Oceanography Division, Scripps Institution of Oceanography, University of California San Diego 8784, La Jolla, California, USA; 2 Microbial and Environmental Genomics, J. Craig Venter Institute, La Jolla, California, USA; Montana State University, Bozeman, Montana, USA

**Keywords:** metabarcoding, amplicon sequencing, 16S, 18S, rRNA, marine microbiology

## Abstract

Mock communities are defined mixtures of cells or DNA for methods development, testing, or validation. Here, we present mock communities based on previously used 16S and 18S rRNA gene sequences from diverse marine microorganisms. Their use as controls for amplicon sequencing enables the detection of biased or aberrant runs.

## ANNOUNCEMENT

Amplicon sequencing, or metabarcoding, of 16S and 18S rRNA genes is widely used to evaluate microbial community composition (1, 2). Low costs and ease of generating data have made such analyses commonplace for wide-ranging studies including those examining marine microorganisms.

Mock communities are defined mixtures comprising plasmids, amplicons, genomic DNA, or whole cells, providing *a priori* knowledge of the abundances of sequences (3). When used as external controls, they help evaluate the bias and accuracy of amplicon sequencing runs (4–8). If a run shows strong bias in the mock communities, environmental sequences from the run may be called into question, as previously observed where a sequencing run completely missed a taxonomic group (9). Without the inclusion of mock communities, errors may not be detected, leading to incorrect conclusions.

With the rising interest in integrating data sets and coordination among laboratories, mock communities are needed to ensure that sequencing runs have an acceptable bias (10, 11). Nevertheless, their inclusion in marine studies is low due to a lack of availability. Mock communities are available from Zymo Research Corporation; however, these do not include marine microorganisms, which are recommended for marine studies (10).

**Fig 1:**
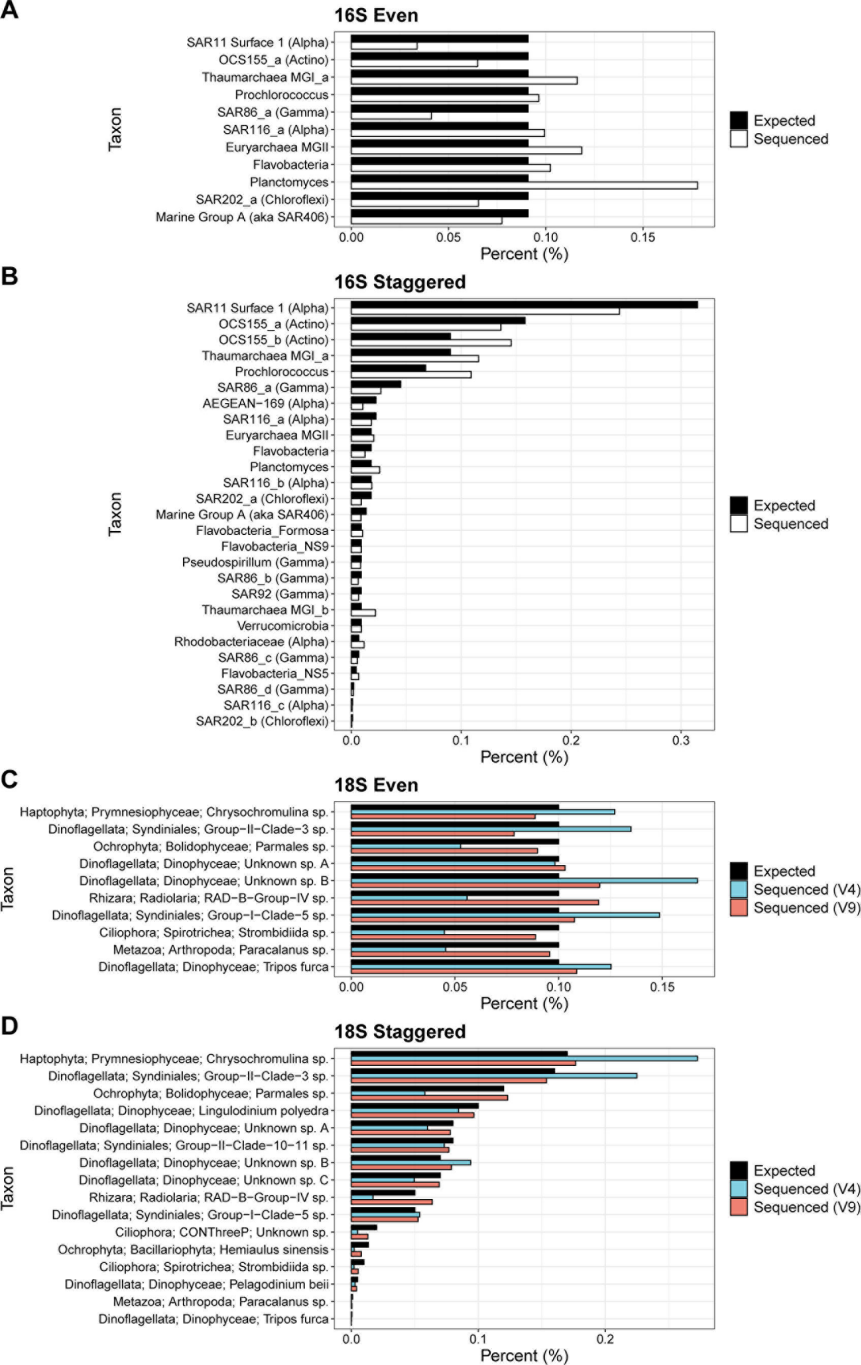
Expected and sequenced relative abundances for the (A) 16S even, (B) 16S staggered, (C) 18S even, and (D) 18S staggered mock communites. 16S taxonomy is from Parada et al. (4), and 18S taxonomy is from the PR^2^ database (13). Expected relative abundances are shown as ratios in the recipe spreadsheets included in the accompanying protocol.

Thus, we constructed mock communities from DNA based on sequences from marine microorganisms. The mock communities largely replicate those previously reported in Parada et al. (4) and Yeh et al. (7), which are now unavailable and no longer able to be recreated. Methods with sequences used are available on protocols.io (12). Briefly, plasmids were synthesized to contain full-length 16S or 18S sequences representing diverse microorganisms (Fig. 1). Sequences were then PCR amplified, quantified, and mixed in either equimolar or different proportions to create even and staggered mock communities, respectively.

For validation, the mock communities were included in amplicon sequencing libraries for the V4–V5 regions of the 16S rRNA gene and both the V4 and V9 regions of the 18S rRNA gene following Rabines et al. (14). Libraries were sequenced on an Illumina NextSeq 2,000 (2 × 300 bp) for the 16S and 18S–V4 libraries or an Illumina MiSeq (2 × 150 bp) for the 18S–V9 library. Samples were then processed in the QIIME2 environment (15). Briefly, sequences were trimmed with cutadapt, denoised with DADA2, and assigned to reference sequences with BLAST (16–18). Certain taxa in all even mock communities deviated from their expected percentages, suggesting potential biases (Fig. 1A and C). Overall, taxa in the staggered communities displayed the expected distribution with some exceptions (Fig. 1B and D). In the 18S communities, the V9 data deviated less than the V4 data, indicating different biases between the two primers.

Further inclusion of these mock communities can facilitate interlaboratory comparisons and ensure that sequencing runs have acceptable bias. They may also be useful for evaluating other primer sets including full-length 16S and 18S sequencing with long-read sequencing. The development of cell-based mock communities would further account for bias during DNA extraction; however, these would be limited to organisms that can be grown and have their own potential bias (3).

## Data Availability

Until there is an adequate repository for this resource, mock communities are available by request to the corresponding author. The sequence data reported in this study have been deposited in the NCBI sequence read archive under the BioProject accession number PRJNA1244892.
